# The management of potential exposures to Cretuzfeld-Jakob Disease (CJD) via endoscopy

**DOI:** 10.1186/1753-6561-5-S6-P312

**Published:** 2011-06-29

**Authors:** A Iten, H Sax, V Camus, D Scalia, A Hadengue, P-Y Martin, S Hurst, D Pittet

**Affiliations:** 1 Infection Control Program, Genève, Switzerland; 2 Service of Gastroenterology, Genève, Switzerland; 3 Committee of ethics , HUG, Genève, Switzerland

## Introduction / objectives

We describe the case of a 68-year-old male with autopsy-confirmed sporadic CJD (CJDs) who had undergone 2 colonoscopies prior to this diagnosis.

The involved endoscopy centre had multiple colonoscopes and gastroscopes that are cleaned and disinfected in the same automatic washers/disinfectors (AWD). There was no system in place to track the use and disinfection of individual endoscopes.

Four questions arise:

- Is it necessary to dispose of colonoscopes potentially contaminated by CJDs?

- Is it necessary to dispose of the AWD where the endoscopes were washed?

- Is it necessary to dispose gastroscopes at risk of contamination during the disinfection process in the AWD?

- Is it necessary to inform the patients who were exposed to these endoscopes ?

## Results

We estimated that this situation occurs approximately 17 times each year in Switzerland. To answer these questions requires data on the presence of CJDs prions in the colon, the risk of contamination of the endoscopes, the risk of prion transmissions to other patients via the endoscopes, and the procedures of cleansing and disinfection. Finally, it is also necessary to take into account psychological, financial and ethical implications for the endoscopy centre and the patients exposed to the potentially contaminated endoscopes.

## Conclusion

This complex situation highlights the need for guidance recommendations in this area.

## Disclosure of interest

None declared.

